# Anticholinergic burden and health-related quality of life among adult patients in a resource-limited setting: a cross-sectional study

**DOI:** 10.1007/s11096-024-01769-z

**Published:** 2024-07-15

**Authors:** Eyob Alemayehu Gebreyohannes, Biniam Siyum Shibe, Wagaye Atalay Taye, Kenneth Lee, Ousman Abubeker Abdela, Emneteab Mesfin Ayele, Eyayaw Ashete Belachew, Segenet Bizuneh Mengistu, Phyo Kyaw Myint, Roy Louis Soiza

**Affiliations:** 1 Quality Use of Medicines and Pharmacy Research Centre, UniSA Clinical and Health Sciences, University of South Australia, Adelaide, Australia; 2 School of Allied Health, The University of Western Australia, Perth, Australia; 3 Institute of Applied Health Sciences, School of Medicine, Medical Sciences and Nutrition, The University of Aberdeen, Aberdeen, UK; 4 Department of Clinical Pharmacy, School of Pharmacy, University of Gondar, Gondar, Ethiopia; 5 Department of Internal Medicine, School of Medicine, University of Gondar, Gondar, Ethiopia

**Keywords:** Anticholinergics, Drug-related side effects and adverse reactions, Health-related quality of life, Medication safety

## Abstract

**Background:**

Anticholinergic medications are now widely acknowledged for their unfavorable risk-to-benefit profile owing to their adverse effects. Health-related quality of life (HRQoL) is commonly regarded as a crucial person-centered outcome.

**Aim:**

This study aimed to investigate the association between anticholinergic burden and HRQoL in hospitalized and ambulatory patients seen in Ethiopia.

**Method:**

This cross-sectional study utilized a questionnaire and medical records to collect data from a convenience sample of adult patients attending both inpatient wards and ambulatory clinic of University of Gondar Comprehensive Specialized Hospital between April and September 2022. Anticholinergic burden was measured by anticholinergic cognitive burdens scale (ACBS), while HRQoL was measured using EQ5D-index (Euroqol-5 dimensions-5-Levels index) and EQ5D-VAS (visual analogue scale). Linear regression was used to assess the influence of high anticholinergic burden (ACBS score ≥ 3) on EQ5D-index and EQ5D-VAS, with adjustments made for sociodemographic and clinical confounders.

**Results:**

A total of 828 patients participated in this study (median (IQR) age was 45.0 (30, 60) and 55.9% were female). On multiple linear regression analysis, high anticholinergic burden was associated with a statistically significant decline in HRQoL, as evidenced by reductions in both EQ5D index (− 0.174 (− 0.250, − 0.098)) and EQ5D-VAS scores (− 9.4 (− 13.3, − 5.2)).

**Conclusion:**

A significant association between high anticholinergic burden and diminished HRQoL was found among a relatively younger cohort in a resource-limited setting, even after adjustment for important confounding variables. Clinicians should be cognizant of the cumulative impact of anticholinergic burden on HRQoL outcomes and strive to minimize anticholinergic burden.

## Impact statements

- The negative association between anticholinergic burden and quality of life highlights the need for careful medication management in resource-limited hospital settings to prevent compromising patients’ quality of life.
- Addressing the high anticholinergic burden through policy changes and enhanced training can lead to improved health outcomes and quality of life for patients in these settings.

## Introduction

Anticholinergic medications are increasingly recognized as having a poor risk-to-benefit ratio [1] due to their association with unpleasant symptoms such as dry mouth and constipation [2] and a range of adverse outcomes, including falls [3], poor cognitive [4] and physical function [5, 6] and even death [7]. The effect of anticholinergic medications is cumulative and numerous tools and scales have been developed to quantify and validate the ‘anticholinergic burden’, however, no one measure is accepted as the gold standard [8]. Despite this, interventions to reduce anticholinergic burden have produced disappointing results, in part due to fear of deprescribing and concerns patients will be worse off without their medications [9].

Health-related quality of life (HRQoL) is often viewed as a key person-centered outcome, as it encompasses both symptoms and outcomes into an overall metric that may be better regarded by clinicians and patients [10]. A recent systematic review and meta-analysis found only four studies measuring the effect of anticholinergic burden on HRQoL [6]. Although all four studies reported high anticholinergic burden was associated with HRQoL, the review found a number of important methodological weaknesses in the included studies, so the evidence was graded as very low, meaning further studies were very likely to change the estimate of the effect size. The main issue was the high risk of bias due to unmeasured confounding from such critical factors as age and comorbidity [6]. This is important because there may be confounding by indication, as anticholinergic medications are used for multiple conditions (e.g. depression, irritable bowel syndrome, overactive bladder) that may themselves worsen HRQoL and accumulate with age [6]. Moreover, there were no studies measuring the effect of anticholinergic burden on HRQoL in a general population in a resource-limited country. This is especially important given the particularly detrimental effects of anticholinergics on older people and the rapidly aging population in many such countries.

This was the first study of its kind in a resource-limited country and would significantly bridge the evidence gap. It was hypothesized that anticholinergic burden would be associated with low HRQoL, even after correction for common confounders such as age and comorbidity.

### Aim

This study therefore aimed to investigate the association between anticholinergic burden and HRQoL in both hospitalized and ambulatory patients seen in Ethiopia, a resource-limited setting.

### Ethics approval

The Institutional Ethical Review Board of the University of Gondar approved the research (VP/RTT/05/359/2021), as did the sponsor of the project, University of Aberdeen School of Medicine, Medical Sciences and Nutrition Ethics Review Board (SERB) (CERB/2021/7/2108). UOGCSH Clinical Directorate granted permission to collect data. Respondents gave both written and verbal consent after being briefed on the study’s objectives.

## Method

### Study design and setting

A cross-sectional study was conducted from April to September 2022 at the University of Gondar Comprehensive and Specialized Hospital (UOGCSH) in-patient wards and ambulatory clinic. This teaching hospital has in-patient and outpatient departments and serves as the only referral center for more than 13 million people in North-west Ethiopia [11].

### Eligibility criteria

Regardless of their medical conditions, all adult patients aged 18 years and above, who were admitted to one of four selected wards (internal medicine, surgical, gynecologic, and psychiatric) or attended the ambulatory clinic at UOGCSH during the study period, were eligible to participate. The exclusion criteria encompassed patients with significant communication impairments, individuals who declined to offer written consent, patients admitted to an intensive care unit, receiving palliative care, or with incomplete medical records.

### Sample size determination and sampling technique

Single population proportion formula with a 5% margin of error and 95% level of significance was used to calculate a minimum required sample size of 384 [12]. In the absence of a prior study in a similar setting, we used a conservative value of 0.5 (50%) for prevalence. A convenience sampling technique was used to recruit the study participants.

### Instruments

HRQoL was assessed using the Euroqol-5 dimensions-5-Levels (EQ-5D-5L) instrument [13]. EQ5D-5L is a generic tool designed to gauge quality of life through five dimensions: mobility, self-care, usual activities, pain and discomfort, and anxiety and depression. Each of these dimensions consists of five levels: no problems, slight problems, moderate problems, severe problems, unable to /extreme problems. By considering all potential combinations across these dimensions, the EQ-5D-5L generates value sets encompassing 5^5^ (= 3125) possibilities, spanning from 11,111 (representing full health) to 55,555 (indicating extreme problems across all dimensions). The EQ-5D-index, specific to the Ethiopian population, was calculated utilizing the EQ-5D-5L value set. This index varies from − 0.718 (reflecting a state worse than death, 55,555) to 1.0 (reflecting full health, 11,111) [14]. Additionally, the EQ-5D-5L includes the EQ-visual analogue scale (EQ-VAS), a vertically oriented scale ranging from 0 (representing the poorest imaginable health) to 100 (representing the best imaginable health).

### Study variables

The outcome variables were EQ-5D-index and EQ-VAS. The independent variable was anticholinergic burden, which was determined by using the Anticholinergic Cognitive Burden Score (ACBS) as described by Boustani and colleagues [15]. Anticholinergic burden is defined as the cumulative exposure to anticholinergic effects resulting from taking one or more medicines possessing anticholinergic properties. High anticholinergic burden was classified as a cumulative score ≥ 3, while low anticholinergic burden was < 3, as assessed by the ACBS [15–17]. Both prescription and over-the-counter medications were taken into account in our anticholinergic burden calculations. Medications used by each patient at the time of data collection were included, drawing from review of their medical records and comprehensive patient interviews. Medications discontinued before the data collection period and new prescriptions issued but not yet initiated by patients at the time of data collection were omitted from the calculations.

Additional variables comprised socio-demographic attributes of the research subjects, such as age, sex, residence, marital status, educational status, and socioeconomic class. Socioeconomic status was determined using an occupation-based classification system [18, 19], where Social Class I encompassed professionals, Class II included managerial and technical roles, Class III comprised skilled workers, Class IV consisted of semi-skilled workers, and Class V included unskilled manual laborers. Other variables included clinical and medicine-related information such as medications each participant was taking, comorbid conditions, Charlson comorbidity index score (CCI, a method for classifying patients’ comorbidities by considering both the quantity and severity of 19 predetermined comorbid conditions [20]), the number of medicines (which was also used to define polypharmacy, the use of ≥ 5 concurrent medicines [21]), and prior hospital admission in the past 30 days.

### Data quality management

Two clinical pharmacy postgraduate students collected the data after being trained on the contents of the instrument, data collection methods, data handling and documentation, and ethical issues. To verify the accuracy of each patient’s medication list, the data collectors carefully cross-checked the information from the patients’ medical records with input from patients, caregivers, treating nurses, and conducted physical checks of the medications where possible. The data collection process was overseen by the one of the investigators (OAA). A pre-test was conducted on 5% of the entire sample, which comprised patients chosen randomly. The information from these patients was then omitted from the final analysis. Throughout the data collection phase, the gathered data was reviewed each day to ensure it was complete, accurate, and consistent.

### Data analysis

All data analyses were conducted using R (version 4.2.2). Descriptive statistics including median, interquartile range (IQR), frequencies and percentages were used to summarize socio-demographic and clinical data. Chi-squared, Fisher’s exact, and Wilcoxon rank-sum tests, as appropriate, were used to determine statistically significant baseline differences between patients with high and low anticholinergic burden. Simple and multiple linear regression were carried out to assess the impact of anticholinergic burden on EQ5D-index and EQ5D-VAS scores. EQ5D index for each participant was calculated by using the *eq5d* function from the package eq5d (version 0.15.1). Continuous variables, age, medication number, CCI, EQ5D-index score, and EQ5D-VAS score were standardized by subtracting each value from its mean and then dividing by its standard deviation. After multiple linear regression analyses, coefficients of EQ5D-index and EQ5D-VAS scores were back transformed to their original scale to facilitate interpretation by multiplying them with their standard deviation. The following variables were initially considered for inclusion into the linear regression models: anticholinergic burden, age, sex, occupation, education, clinical setting, history of admission in the past 30 days, number of medications, CCI, infectious and parasitic diseases, neoplasms, endocrine, nutritional and metabolic diseases, diseases of the digestive system, mental and behavioral disorders, diseases of the blood, and diseases of the genitourinary system. The variance inflation factor (VIF) was utilized to assess multicollinearity, leveraging the *vif* function from the car package (version 3.1.2). In our analysis, variables demonstrating *p*-values < 0.2 in simple linear regression were deemed candidates for inclusion in the multiple linear regression model [22, 23]. Despite an initial p-value exceeding 0.2, we opted to include age in the final EQ5D-index model due to its established association with EQ5D-index as reported in a previous study [24]. The *stepAIC* function from the package MASS (version 7.3.60) was used to select variables for the multiple regression model through backward elimination. Akaike information criterion (AIC) was used to determine the final models.

## Results

A total of 828 patients participated in this study. The median (IQR) age of the participants was 45.0 (30, 60) years. More than half of participants were female (n = 463, 55.9%), and two-third (n = 553, 66.7%) of the participants were married. Most participants were urban dwellers (n = 466, 56.3%) and Class V workers (n = 510, 61.6%). Almost half of the participants (n = 419, 50.6%) were hospitalized, and the other half (n = 409, 49.4%) attended the ambulatory clinic. Compared to patients that had low anticholinergic burden group, those with high anticholinergic burden had higher median number of medications and lower EQ5D-index and EQ5D-VAS scores (Table 1).

**Table 1:** Sociodemographic and clinical characteristics of study participants

Characteristics		Low anticholinergic burden (ACBS < 3) n = 752	High anticholinergic burden (ACBS ≥ 3) n = 76	Total n = 828	*p*-value
*Sociodemographic*					
Age—median (IQR)		45 (30, 60)	50 (35, 63.2)	45 (30, 60)	0.144^α^
Sex–n (%)	Male	337 (44.8%)	28 (36.8%)	365 (44.1%)	0.225^β^
	Female	415 (55.2%)	48 (63.2%)	463 (55.9%)	
Residency – n (%)	Urban	420 (55.9%)	46 (60.5%)	466 (56.3%)	0.508^β^
	Rural	332 (44.1%)	30 (39.5%)	362 (43.7%)	
Marital status – n (%)	Married	501(66.6%)	51 (67.1%)	553 (66.7%)	0.999^β^
	Single/ Divorced/ Widowed	251 (33.4%)	25 (32.9%)	276 (33.3%)	
Education – n (%)	No formal education	355 (47.2%)	42 (55.3%)	399 (47.9%)	0.406^β^
	Primary education	167 (22.2%)	14 (18.4%)	181 (21.9%)	
	Secondary/ tertiary education	230 (30.6%)	20 (26.3%)	250 (30.2%)	
Occupation – n (%)	Class I to IV	290 (38.6%)	28 (36.8%)	318 (38.4%)	0.865^β^
	Class V	462 (61.4%)	48 (63.2%)	510 (61.6%)	
*Clinical*					
Setting	Inpatient	382 (50.8%)	37 (48.7%)	419 (50.6%)	0.817^β^
	Outpatient	370 (49.2%)	39 (51.3%)	409 (49.4%)	
CCI – median (IQR)		1 (0, 3)	1 (1, 3)	1 (0, 3)	0.109^α^
Comorbidity – n (%)	Infectious and parasitic diseases	196 (26.1%)	17 (22.4%)	214 (25.7%)	0.572^β^
	Diseases of the circulatory system	301 (40.0%)	31 (40.8%)	332 (40.1%)	0.995^β^
	Endocrine, nutritional and metabolic diseases	140 (18.6%)	15 (19.7%)	155 (18.7%)	0.933^β^
	Diseases of the digestive system	99 (13.2%)	1 (1.3%)	100 (12.1%)	** < 0.005**^**γ**^
	Diseases of the respiratory system	26 (3.5%)	1 (1.3%)	27 (3.3%)	0.502^γ^
	Mental and behavioral disorders	15 (2.0%)	12 (15.8%)	27 (3.3%)	** < 0.001**^**γ**^
	Diseases of the blood	57 (7.6%)	2 (2.6%)	59 (7.1%)	0.173^β^
Number of medications—median (IQR)		3 (2, 4)	4 (3, 5.25)	3 (2, 4)	** < 0.001**^**α**^
Polypharmacy–n (%)	Yes	178 (23.7%)	28 (36.8%)	206 (24.9)	**0.017**^**β**^
Prior admission within 30 days–n (%)	Yes	57 (7.6%)	3 (3.9%)	60 (7.2%)	0.351^β^
EQ5D VAS—median (IQR)		70 (50, 80)	60 (50, 70)	70 (50, 80)	** < 0.001**^**α**^
EQ5D index—median (IQR)		0.78 (0.51, 0.90)	0.63 (0.51, 0.83)	0.77 (0.49, 0.89)	** < 0.001**^**α**^

Bold indicates statistical significance

*ACBS* Anticholinergic Cognitive Burden Score, *CCI* Charlson comorbidity index score, *IQR* Interquartile range

^α^The Wilcoxon rank-sum test

^β^Chi-squared test

^γ^Fisher’s exact test

### Frequency of medications possessing anticholinergic activity

**Fig. 1:**
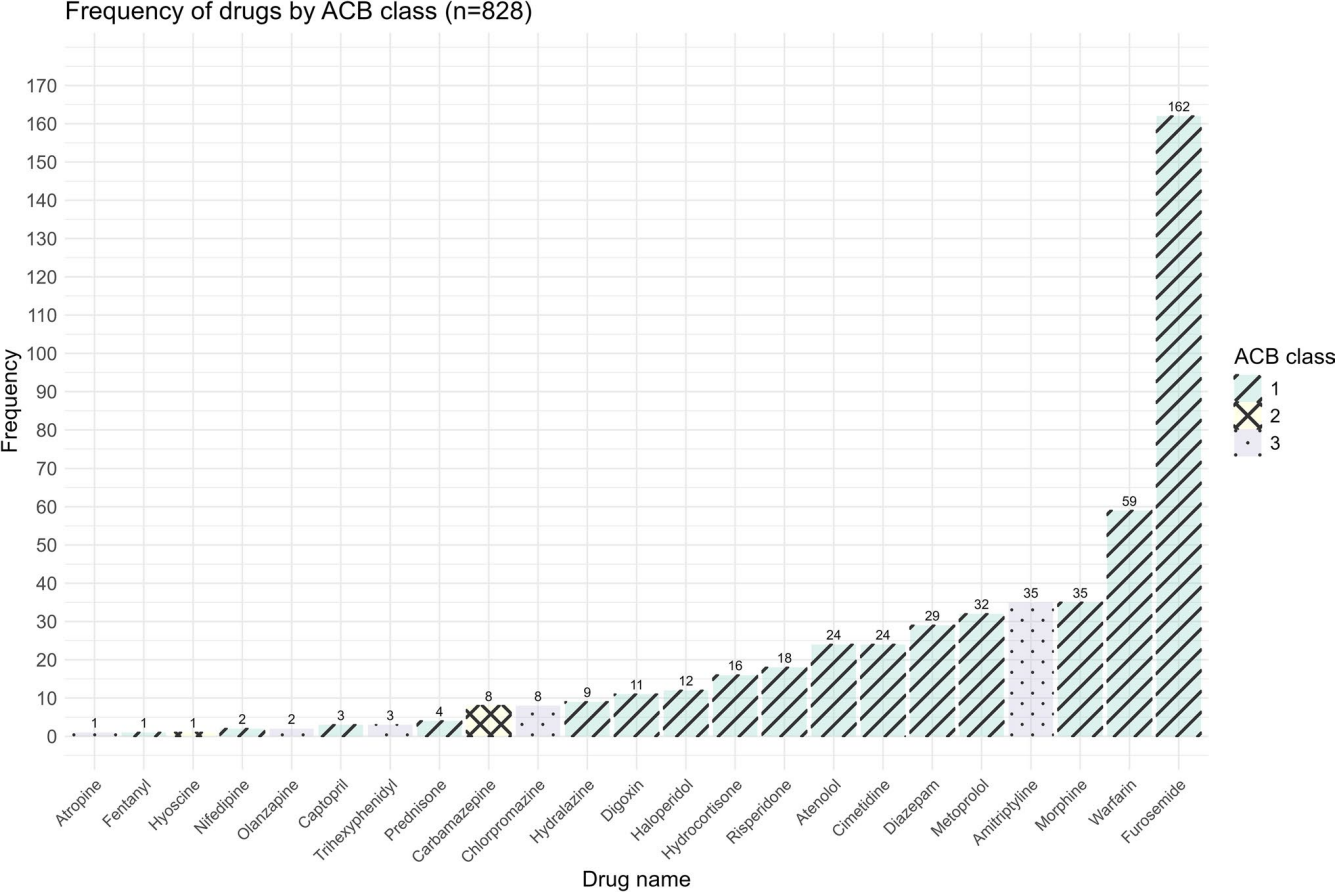
Frequency of medications with anticholinergic activity by anticholinergic burden class. *ACB* Anticholinergic burden

Most of these medications (n = 16) with anticholinergic activity had an anticholinergic burden score of 1. Of these, furosemide, warfarin, and morphine were the three most frequently used medications. Two medications, carbamazepine and hyoscine, had an anticholinergic burden score of 2, while five medications, amitriptyline, chlorpromazine, trihexyphenidyl, olanzapine, and atropine had a score of 3 (Fig. 1).

### Anticholinergic burden and HRQoL

Overall, the majority of participants reported no-to-slight problems on mobility (n = 469, 56.7%), self-care (n = 610, 73.7%), and anxiety/depression domains (n = 590, 71.3%), and moderate-to-extreme problems in the usual activities (n = 492, 59.3%) and pain/discomfort domains (n = 458, 55.3%). Compared to patients that had low anticholinergic burden group, those with high anticholinergic burden had more participants with moderate-to-extreme problems in the self-care, pain/discomfort, and anxiety/depression domains. No statistically significant difference was observed among patients with low and high anticholinergic burden in the mobility and usual activities domains (Table 2).

**Table 2:** EQ-5D domains stratified by the anticholinergic burden

		Low anticholinergic burden (ACBS < 3) n = 752	High anticholinergic burden (ACBS ≥ 3) n = 76	Total n = 828	*p*-value
Mobility	No problems	247 (32.8%)	17 (22.4%)	264 (31.9%)	0.182^γ^
	Slight problems	187 (24.9%)	18 (23.7%)	205 (24.8%)	
	Moderate problems	192 (25.5%)	22 (28.9%)	214 (25.8%)	
	Severe problems	99 (13.2%)	14 (18.4%)	113 (13.6%)	
	Unable	27 (3.6%)	5 (6.6%)	32 (3.9%)	
Self-care	No problems	365 (48.5%)	19 (25.0%)	384 (46.4%)	** < 0.001**^**γ**^
	Slight problems	205 (27.3%)	21 (27.6%)	226 (27.3%)	
	Moderate problems	108 (14.4%)	20 (26.3%)	128 (15.5%)	
	Severe problems	62 (8.2%)	11 (14.5%)	73 (8.8%)	
	Unable	12 (1.6%)	5 (6.6%)	17 (2.1%)	
Usual activities	No problems	163 (21.7%)	8 (10.5%)	171 (20.7%)	0.097^β^
	Slight problems	150 (19.9%)	15 (19.7%)	165 (19.9%)	
	Moderate problems	117 (15.6%)	15 (19.7%)	132 (15.9%)	
	Severe problems	157 (20.9%)	23 (30.3%)	180 (21.7%)	
	Unable	165 (21.9%)	15 (19.7%)	180 (21.7%)	
Pain/ Discomfort	No pain	143 (19.0%)	8 (10.5%)	151 (18.2%)	**0.001**^**γ**^
	Slight pain	207 (27.5%)	12 (15.8%)	219 (26.4%)	
	Moderate pain	253 (33.6%)	27 (35.5%)	280 (33.8%)	
	Severe pain	139 (18.5%)	25 (32.9%)	164 (19.8%)	
	Extreme pain	10 (1.3%)	4 (5.3%)	14 (1.7%)	
Anxiety/ Depression	Not anxious/depressed	348 (46.3%)	18 (23.7%)	366 (44.2%)	** < 0.001**^**γ**^
	Slightly anxious/depressed	206 (27.4%)	18 (23.7%)	224 (27.1%)	
	Moderately anxious/depressed	143 (19.0%)	21 (27.6%)	164 (19.8%)	
	Severely anxious/depressed	49 (6.5%)	17 (22.4%)	66 (8.0%)	
	Extremely anxious/depressed	6 (0.8%)	2 (2.6%)	8 (1.0%)	

Bold indicates statistical significance

*ACBS* Anticholinergic cognitive burden score

^β^Chi-squared test

^γ^Fisher’s exact test

On multiple linear regression analysis, high anticholinergic burden was associated with a statistically significant decline in HRQoL, as evidenced by reductions in both EQ5D index and EQ5D-VAS scores. The findings indicates that, high anticholinergic burden was associated with a decline of 9.4 in the EQ5D-VAS score (β (95%CI): -0.520 (-0.734, -0.287) multiplied by the SD of EQ5D-VAS 18.08) (Table 3). Similarly, compared to patients with low anticholinergic burden, patients exposed to high anticholinergic burden had an EQ5D index score that was lower by 0.174 (β (95% CI): -0.534 (-0.768, -0.301) multiplied by the SD of EQ5D-VAS 0.325) (Table 4).

**Table 3:** Association between anticholinergic burden and EQ-5D VAS score

Variables*		Simple linear regression		Multiple linear regression	
		β (95% CI)	*p*-value	β (95% CI)	*p*-value
Anticholinergic burden	High	− 0.520 (− 0.734, − 0.287)	** < 0.001**	− 0.480 (− 0.696, − 0.265)	** < 0.001**
Sex	Male	− 0.120 (− 0.017, 0.257)	0.086	0.163 (0.039, 0.288)	**0.010**
Occupation	Class V	− 0.492 (− 0.628, − 0.356)	** < 0.001**	− 0.224 (− 0.379, − 0.07)	**0.005**
Education	Primary	0.198 (0.024, 0.372)	**0.026**	0.193 (0.034, 0.352)	**0.017**
	Secondary or tertiary	0.363 (0.207, 0.520)	** < 0.001**	0.039 (− 0.124, 0.202)	0.638
Clinical setting	Outpatient	0.618 (0.488, 0.748)	** < 0.001**	0.268 (0.109, 0.426)	**0.001**
Prior admission		0.856 (0.599, 1.113)	** < 0.001**	0.504 (0.267, 0.741)	** < 0.001**
Number of medications		− 0.397 (− 0.460, − 0.335)	** < 0.001**	− 0.19 (− 0.263, − 0.116)	** < 0.001**
CCI		− 0.223 (− 0.289, − 0.156)	** < 0.001**	− 0.142 (− 0.207, − 0.077)	** < 0.001**
Endocrine, nutritional and metabolic diseases		0.372 (0.198, 0.545)	** < 0.001**	0.183 (0.027, 0.34)	**0.022**
Mental and behavioral disorders		0.285 (− 0.099, 0.668)	0.146	0.406 (0.045, 0.767)	**0.027**
Diseases of the blood		− 0.496 (− 0.759, − 0.233)	** < 0.001**	− 0.29 (− 0.528, − 0.052)	**0.017**
Diseases of the genitourinary system		− 0.844 (− 1.117, − 0.571)	** < 0.001**	− 0.387 (− 0.636, − 0.139)	**0.002**

EQ5D-VAS score, number of medications and CCI were standardized. Bold indicates statistical significance

*CCI* Charlson comorbidity index score

*The comparator groups for categorical variables are defined as presented in Table 1

**Table 4:** Association between anticholinergic burden and EQ-5D index score

Variables*		Simple linear regression		Multiple linear regression	
		β (95% CI)	*p*-value	β (95% CI)	*p*-value
Anticholinergic burden	High	− 0.534 (− 0.768, − 0.301)	** < 0.001**	− 0.403 (− 0.603, − 0.202)	** < 0.001**
Age		− 0.014 (− 0.082, 0.054)	0.691	0.064 (− 0.023, 0.152)	0.151
Occupation	Class V	− 0.505 (− 0.641, − 0.369)	** < 0.001**	− 0.159 (− 0.304, − 0.014)	**0.032**
Marital status	never married/ divorced/ widowed	− 0.193 (− 0.337, − 0.048)	**0.009**	− 0.231 (− 0.356, − 0.106)	** < 0.001**
Education	Primary	0.200 (0.025, 0.374)	**0.025**	0.292 (0.134, 0.45)	** < 0.001**
	Secondary or tertiary	0.335 (0.178, 0.492)	** < 0.001**	0.089 (− 0.074, 0.252)	0.285
Clinical setting	Outpatient	0.851 (0.727, 0.974)	** < 0.001**	0.514 (0.364, 0.664)	** < 0.001**
Prior admission		0.788 (0.530, 1.046)	** < 0.001**	0.292 (0.063, 0.521)	**0.012**
Number of medications		− 0.435 (− 0.497, − 0.374)	** < 0.001**	− 0.22 (− 0.289, − 0.15)	** < 0.001**
CCI		− 0.148 (− 0.215, − 0.080)	** < 0.001**	− 0.121 (− 0.2, − 0.041)	**0.003**
Endocrine, nutritional and metabolic diseases		0.470 (0.297, 0.642)	** < 0.001**	0.223 (0.073, 0.373)	**0.004**
Diseases of the genitourinary system		− 0.772 (− 1.046, − 0.499)	** < 0.001**	− 0.321 (− 0.56, − 0.082)	**0.008**

EQ5D-index score, age, number of medications, and CCI were standardized. Bold indicates statistical significance

*CCI* Charlson comorbidity index score

*The comparator groups for categorical variables are defined as presented in Table 1

## Discussion

### Statement of key findings

Our study shows for the first time an association between high anticholinergic burden and poor HRQoL in a resource-limited country. The association remained strongly statistically significant even after correction for important potential confounders, such as age, co-morbidity burden and number of medications. Our findings substantially confirm and build upon the previously published evidence that anticholinergic medications have an adverse effect on HRQoL [6]. This adverse effect was seen even in a much more ethnically diverse and significantly younger population than has ever been previously studied, and remained even after adjustment for confounders that were not accounted for in previous studies.

### Strengths and weaknesses

Our study has a number of limitations. Firstly, like any observational study, causality cannot be inferred. This was mitigated against by correcting for major confounders, but residual confounding can never be fully excluded. Secondly, a convenience sample from inpatient and outpatient settings at a single hospital was used, so results may not be generalizable to the wider Ethiopian population, nor that of other resource-limited countries. However, by choosing both multiple inpatient wards and an outpatient clinic within the major tertiary referral centre in the area, we believe the sample is reasonably representative of the healthcare-seeking population of Ethiopia. Thirdly, our measure of exposure relied on a single measure of anticholinergic burden at a single timepoint, without taking into consideration either duration of exposure or dosage. Nevertheless, the ACBS at a single time-point is the most commonly used scale in the literature [6] and the clear association with HRQOL suggests this was not an important flaw. Lastly, there is disparity in the number of patients between high (76 patients) and low (752 patients) anticholinergic burden. However, this is due to the cumulative nature of anticholinergic burden and the relatively younger study population, which is less intensively medicated. The study has some important strengths, with a robust process of medicine reconciliation from at least two different sources, ensuring that the ACBS was accurate.

### Interpretation

In the only published systematic review of the effects of anticholinergic medications on HRQoL, Stewart et al. [6] found only four studies including just 2635 people. High anticholinergic burden was associated with low HRQoL but firm conclusions could not be made due to heterogeneity in study populations, differences in ways both anticholinergic burden and HRQoL were measured, and because the risk of bias was medium to high. Furthermore, the studies were all conducted either in North America or Australia and two considered only special populations (dying patients under palliative care and those with dementia). Since then, two more studies have been published that also found an association between high anticholinergic burden and low HRQoL, a cohort study in the UK [25] and a study of patients with myeloma in a clinic in Brazil [26]. Despite the consistency of the finding, there are no studies measuring the effect of anticholinergic burden on HRQoL in a general population in a resource-limited country. Moreover, our study addressed many of the limitations identified by the systematic review and meta-analysis, with a more appropriate statistical analysis plan and correction for potentially important confounding factors. Taken together with the result of the two most recent studies, there is a clear message that anticholinergic medications worsen HRQoL.

The simplest explanation is arguably that the adverse effects of antimuscarinic blockade directly contribute to worsening HRQoL. Dry mouth is the most commonly reported side effect [2], but other common adverse effects include constipation, urinary retention, blurred vision, cognitive impairment, dizziness and postural instability, heat intolerance and sleep disturbance [27, 28]. However, it is also possible that some or all of the association is due to secondary consequences of taking anticholinergic medications. For example, the same systematic review by Stewart et al. found better evidence that anticholinergic burden worsens physical function than it did for HRQoL, but clearly one could lead to the other. The observed association with HRQoL could also be driven by the increased risk of falls or dementia [3, 4]. A final explanation is that there is a degree of confounding by indication. However, this is unlikely given the richness of comorbidity data in our dataset and the CCI is widely used to measure the burden of disease. Further research would be required to investigate what exactly drives the association.

### Further research

Our results add to the wealth of evidence that clinicians should maximize their efforts to minimize anticholinergic burden in their patients. This is already recommended by several bodies for specific groups such as in older people by the American Association of Family Physicians [29], or in those with dementia by the National Institute for Health and Care Excellence in the National Health Service (NHS) in England [30]. However, our findings suggest that this advice could be extended to the wider population, as is already advised by the NHS in Scotland in their polypharmacy guidance [31]. However, this may be easier said than done. A systematic review and meta-analysis of trials of interventions to reduce anticholinergic burden found these interventions were usually unsuccessful in reducing anticholinergic burden scores. The interventions included a multi-disciplinary approach to reviewing medications and deprescribing anticholinergic drugs [9]. Given this failure, it was no surprise that the interventions as a whole did not have an impact of quality of life, or any of the other outcome measures, such as cognition. Only two trials sought to measure the impact of de-prescribing interventions on HRQoL. A community pharmacist-led intervention in the Netherlands failed to have any impact on either anticholinergic burden or HRQoL [32]. A trial of the Multidisciplinary Multistep Medication Review in 59 Dutch nursing homes successfully reduced inappropriate medications and improved HRQoL despite failing to demonstrate a statistically significant reduction in anticholinergic burden [33]. Previous studies have identified barriers to reducing anticholinergic burden such as poor communication between healthcare professionals and patients, as well as uncertainties about the value of reducing anticholinergic burden [34]. Successful interventions may require clear guidance, training, continued engagement between the patient and clinician and shared decision-making, with a focus on person-centered care [9].

## Conclusion

In conclusion, this study highlights a significant association between high anticholinergic burden and diminished HRQoL among a relatively younger cohort in a resource-limited setting, even after adjustment for important confounding variables. These findings highlight the imperative of reducing anticholinergic burden, particularly in patients taking multiple medications, emphasizing the cumulative impact of anticholinergic burden on HRQoL. Future studies should delve deeper into the longitudinal effects of anticholinergic burden on HRQoL, exploring potential interventions to mitigate its adverse impact.
