# Correction: Anticholinergic burden and health‑related quality of life among adult patients in a resource‑limited setting: a cross‑sectional study

**DOI:** 10.1007/s11096-026-02182-4

**Published:** 2026-07-03

**Authors:** Eyob Alemayehu Gebreyohannes, Biniam Siyum Shibe, Wagaye Atalay Taye, Kenneth Lee, Ousman Abubeker Abdela, Emneteab Mesfin Ayele, Eyayaw Ashete Belachew, Segenet Bizuneh Mengistu, Phyo Kyaw Myint, Roy Louis Soiza

**Affiliations:** 1 Quality Use of Medicines and Pharmacy Research Centre, UniSA Clinical and Health Sciences, University of South Australia, Adelaide, Australia; 2 School of Allied Health, The University of Western Australia, Perth, Australia; 3 Institute of Applied Health Sciences, School of Medicine, Medical Sciences and Nutrition, The University of Aberdeen, Aberdeen, UK; 4 Department of Clinical Pharmacy, School of Pharmacy, University of Gondar, Gondar, Ethiopia; 5 Department of Internal Medicine, School of Medicine, University of Gondar, Gondar, Ethiopia

**Correction to: International Journal of Clinical Pharmacy (2024) 46:1352–1361** 10.1007/s11096-024-01769-z

In this article, the descriptive frequency counts in subsection *“Frequency of medications possessing anticholinergic activity”* under the Results section were published incorrectly.

The sentence incorrectly written as: Most of these medications (n = 15) with anticholinergic activity had an anticholinergic burden score of 1. Of these, furosemide, warfarin, and morphine were the three most frequently used medications. Two medications, carbamazepine and hyoscine, had an anticholinergic burden score of 2, while four medications, amitriptyline, chlorpromazine, olanzapine, and atropine had a score of 3 (Fig. 1).

The correct sentence should read: Most of these medications (n = 16) with anticholinergic activity had an anticholinergic burden score of 1. Of these, furosemide, warfarin, and morphine were the three most frequently used medications. Two medications, carbamazepine and hyoscine, had an anticholinergic burden score of 2, while five medications, amitriptyline, chlorpromazine, trihexyphenidyl, olanzapine, and atropine had a score of 3 (Fig. 1).

Additionally, Figure 1 was appeared incorrectly but has now been corrected in the original publication. For completeness and transparency, the correct and old incorrect versions are displayed below.

The original article has been corrected.

Incorrect figure 1

**Fig. 1:**
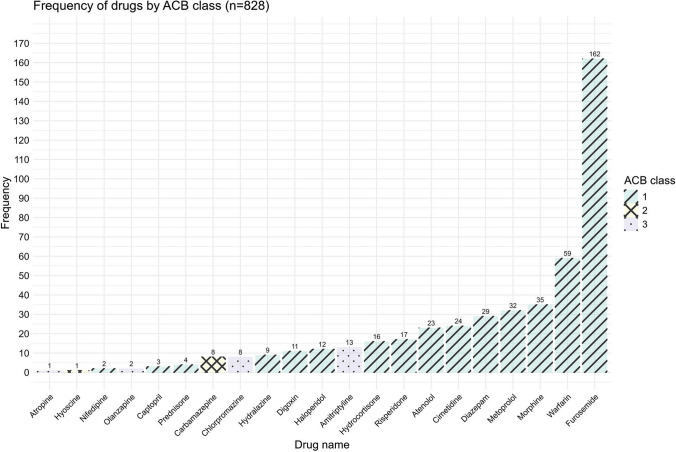
Frequency of medications with anticholinergic activity by anticholinergic burden class. *ACB* Anticholinergic burden

Correct figure 1

**Fig. 1:**
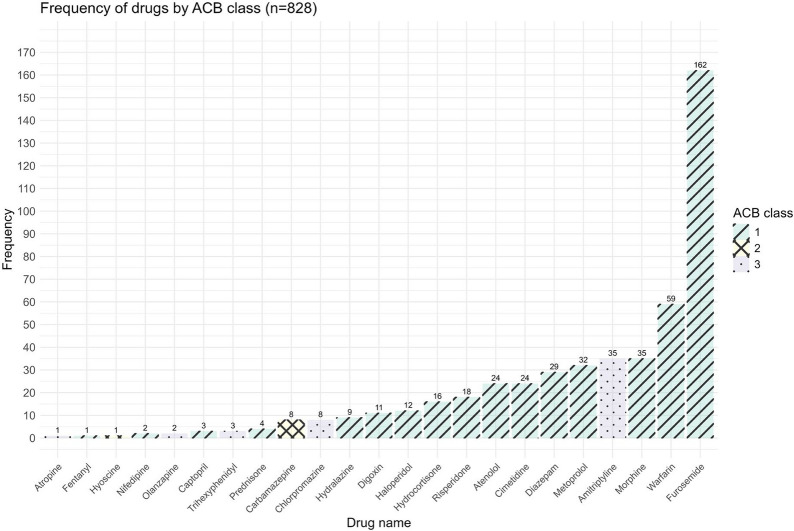
Frequency of medications with anticholinergic activity by anticholinergic burden class. *ACB* Anticholinergic burden

